# SaeboGlove therapy for upper limb disability and severe hand impairment after stroke (SUSHI): Study protocol for a randomised controlled trial

**DOI:** 10.1177/23969873211036586

**Published:** 2021-08-01

**Authors:** Jen Alexander, Peter Langhorne, Lisa Kidd, Olivia Wu, Alex McConnachie, Frederike van Wijck, Jesse Dawson

**Affiliations:** 1 Institute of Cardiovascular and Medical Sciences, NHS Greater Glasgow and Clyde, Queen Elizabeth University Hospital, Glasgow, UK; 2 Academic Section of Geriatric Medicine, Institute of Cardiovascular and Medical Sciences, College of Medical, Veterinary & Life Sciences, University of Glasgow, Royal Infirmary, Glasgow, UK; 3 School of Medicine, Dentistry & Nursing, College of Medical, Veterinary & Life Sciences, University of Glasgow, Glasgow, UK; 4 Health Economics and Health Technology Assessment, Institute of Health & Wellbeing, University of Glasgow, Glasgow, UK; 5 Robertson Centre for Biostatistics, Institute of Health and Wellbeing, University of Glasgow, Glasgow, UK; 6 Life Sciences, 3525 Glasgow Caledonian University, Glasgow, UK; 7 Institute of Cardiovascular and Medical Sciences, College of Medical, Veterinary & Life Sciences, University of Glasgow, Queen Elizabeth University Hospital, Glasgow, UK

**Keywords:** Stroke, upper limb, rehabilitation, dynamic hand orthosis, randomised controlled trial

## Abstract

**Background:**

Impaired active digital extension is common after stroke, hindering functional rehabilitation, and predicting poor recovery. The SaeboGlove assists digital extension and may improve outcome after stroke. We recently performed a single group, open, pilot trial of the SaeboGlove early after stroke which demonstrated satisfactory safety, feasibility and acceptability. An adequately powered randomised clinical trial is now needed to assess the clinical effectiveness of the SaeboGlove.

**Methods:**

SUSHI is a pragmatic, multicentre, parallel-group, randomised controlled trial with blinded outcome assessment, and embedded process and economic evaluations. Adults, 7–60 days post-stroke, with upper limb disability and severe hand impairment, including reduced active digital extension, will be recruited from NHS inpatient stroke services in Scotland. Participants will be randomised on a 1:1 basis to receive 6 weeks of self-directed, repetitive, functional-based practice involving a SaeboGlove plus usual care, or usual care only. The primary outcome is upper limb function measured by the Action Research Arm Test (ARAT) at 6 weeks. Secondary outcomes will be measured at 6 and 14 weeks. A process evaluation will be performed via interviews with ‘intervention’ participants, and their carers and clinical therapists. A within-trial cost-effectiveness analysis will be performed. 110 participants are required to detect a difference between groups of 9 in the ARAT with 90% power at a 5% significance level allowing for 11% attrition.

**Discussion:**

SUSHI will determine if SaeboGlove self-directed, repetitive, functional-based practice improves upper limb function after stroke, whether it is acceptable to stroke survivors and whether it is cost-effective.

## Background

Stroke is the third leading cause of disability worldwide.^1^ Upper limb motor impairment contributes significantly to the burden experienced by stroke survivors. It affects around 80% of people with stroke^2^ and approximately 50% of them have no improvement in upper limb function six months post-stroke.^3^ Long-term upper limb impairment is associated with increased disability^4^ and reduced quality of life.^5^

Impaired active digital extension is the most common upper limb motor impairment after stroke.^6^ It reduces hand opening essential for upper limb function^7^ and predicts poor upper limb recovery.^8^ Repetitive, functional-based rehabilitation and self-practice activities are advocated by national clinical guidelines^9,10^ and systematic review evidence.^11–13^ However, lack of active digital extension can prevent people accessing guideline-based activities, both in clinical^14,15^ and research^16^ settings, and no evidence-based interventions have been identified to improve this.^3,13,17^ This may account for the particularly poor upper limb prognosis experienced after stroke.

**Figure 1.**
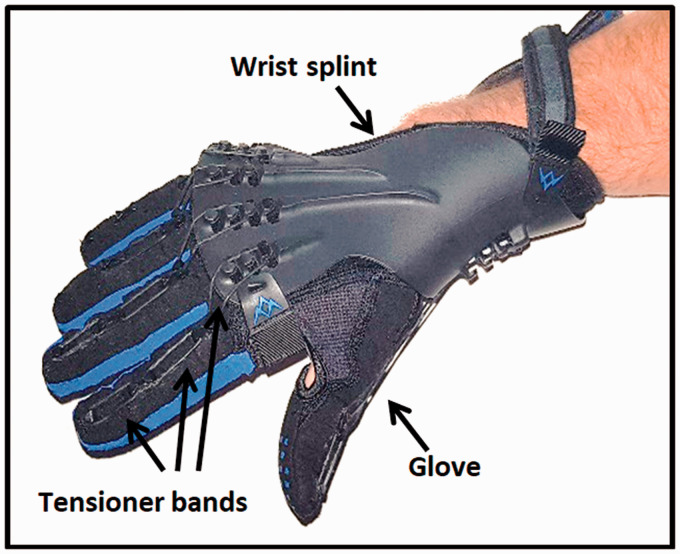
The SaeboGlove. The SaeboGlove consists of a glove velcroed inside a wrist splint, and bands of different size that offer variable extensor assistance. This assistance increases hand opening when it is limited, improving access to recommended rehabilitation activities.

The SaeboGlove is a CE marked mechanical hand orthosis already used in some health services. It consists of a glove, fixed using velcro inside a wrist splint (Figure 1),^18^ and offers digital extensor assistance using tensioner bands which span between hooks over weak joints to enable hand opening.^18^ Consequently, it improves access to repetitive, functional-based upper limb rehabilitation and self-practice opportunities,^19^ regardless of extensor weakness severity. This might promote engagement in recommended rehabilitation activities and improve long-term recovery; addressing key priorities in stroke care.

We recently completed a single-group pilot trial of four weeks of SaeboGlove self-directed, repetitive, functional-based practice in people with reduced active digital extension early after stroke (n = 12, mean (range) 27 (4–80) days post).^19^ The intervention was found to be safe, feasible and acceptable. We used the revised, standardised^20^ and validated^21^ version of the original ARAT by Lyle^22^ (scale 0–57) which does not specify time limits. As this test has been historically described as a measure of upper limb function, a term that aligns with upper limb activity capacity within The International Classification of Functioning, Disability and Health model, this manuscript will continue to use this terminology. ARAT scores improved by a mean (SD) of 18.8 (13.5) points.^19^ This improvement is nearly double that observed in historical controls (10 (15) points)^23^ but the absence of a control group limits interpretation.

In this protocol paper, we describe the SUSHI trial. The primary objective of the SUSHI trial is to assess the clinical effectiveness of SaeboGlove self-directed, repetitive, functional-based practice plus usual care when compared to usual care alone in people with upper limb disability and severe hand impairment, including reduced active digital extension after recent stroke. We hypothesise that upper limb function (ARAT) will be significantly greater in the intervention group immediately post intervention.

## Methods

### Trial design

**Figure 2.**
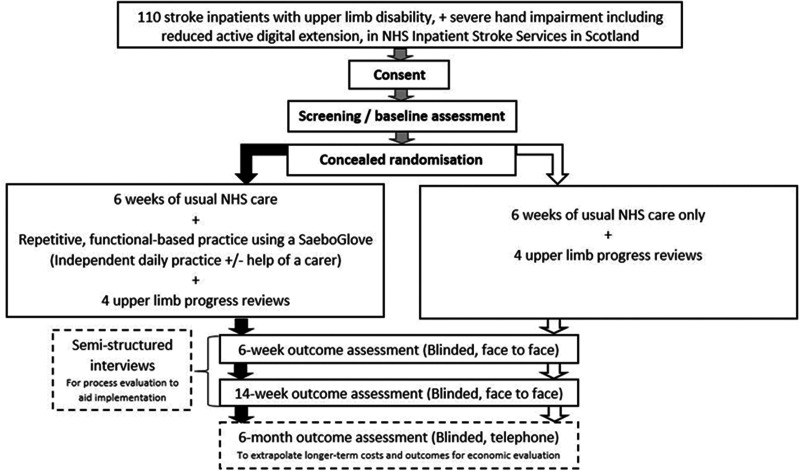
Overview of methods used for RCT, and process and economic evaluations.

The SUSHI trial is a pragmatic, multicentre, parallel-group, RCT comparing self-directed, repetitive, functional-based practice with a SaeboGlove plus usual care, to usual care alone. The trial includes blinded outcome assessments with embedded process and economic evaluation. Follow-up for primary and secondary outcomes will occur at 6 and 14 weeks post randomisation. A further follow-up will be performed at 6 months to inform assessment of long-term benefits and economic evaluation. Figure 2 provides an overview of the SUSHI trial. The study is presented according to the Standard Protocol Items: Recommendations for Interventional Trials (SPIRIT)^24^ and a summary is provided in Table 1.

**Table 1.** Standard protocol items.

Trial activity	Study period					
	Enrolment	Baseline assessment (≤48 hr after screening)	Intervention	Outcome assessments		
	Pre week 0	Week 0	Weeks 1–6	Week 6	Week 14	Month 6
Written informed consent	X					
Contact details	X					
Demographics	X					
Stroke details	X					
Relevant comorbidities	X					
Hand + Wrist movement (Active, and passive (contractures + spasticity)	X					
Hand impairment (Hand sub-section of Fugl Meyer upper extremity scale)	X	*				
Upper limb function (Action Research Arm Test)	X	*		X*	X	
Screening check list	X					
Current upper limb treatment		X				
Upper limb impairment (Fugl Meyer upper extremity scale)		X		X	X	
Habitual functional use (Motor Activity Log)		X		X	X	X
Degree of disability (Modified Rankin Scale)		X		X	X	X
Activities of daily living (Barthel Index)		X		X	X	
Quality of life						
(EQ-5D-5L)		X		X	X	X
(Stroke Impact Scale)		X		X	X	
Upper limb pain intensity (Visual analogy scale)		X		X	X	
NHS and social services resource use questionnaire		X			X	X
Randomisation		X				
Adverse events			X	X	X	
SaeboGlove therapy plus usual care			X (Intervention group)			
Usual care**			X (Control group)			
Rehabilitation booklet completed, weekly telephone reminder			X	X		
Usual care recorded			X			

Recommendations for Interventional Trials (SPIRIT): Schedule of enrolment, interventions, assessments and visits. EQ-5D-5L: EuroQol with 5 Dimensions and 5 Levels, UL: Upper limb.

* Hand sub-section and ARAT only repeated if baseline assessment occurs >48 hours after the screening assessment or if it occurred <48 hours ago and assessor is concerned that eligibility may have changed. * represents the primary outcome. ** Optional 6 weeks of SaeboGlove therapy offered after 14 week outcome visit is completed.

### Trial setting

Participants will be recruited within NHS Inpatient Stroke Services in Scotland.

### Trial status

The study is registered on ClinicalTrials.gov (NCT04007315, 5 July 2019). Participant recruitment started in November 2019 (protocol version 2.0, 15^th^ July 2019, current 3.0, 14^th^ April 2020). Recruitment was suspended in March 2020 due to Covid-19 after 16 participants were randomised. Enrolment began again in July 2020 and is expected to close in July 2022.

### Ethical/regulatory approval

The study sponsor is the NHS GG&C Health Board. The study will be performed in line with the Declaration of Helsinki. Ethical approval was granted by the West of Scotland Research Ethics Committee (REC) 1 in August 2019 (19/WS/0097). NHS Board approvals were granted by NHS GG&C (GN19ST318) and NHS Lanarkshire (L19046) in November 2019. At the time of writing approval from other sites is awaited. The Principal Investigator at each site will be notified of protocol amendments. The SaeboGlove is CE marked for use as a rehabilitation device so prior regulatory approval from the MHRA is not required.

### Eligibility criteria

Participants aged 18 years or older with a new clinical stroke diagnosis that occurred 7–60 days prior to enrolment will be included in the study if they have upper limb disability (ARAT ≤46) and severe hand impairment (Fugl Meyer Upper Extremity scale (FMUE) hand sub-section ≤7, and reduced active digital extension). The ARAT range was based on feasibility data and the limited function in this group.^25^ A full list of the inclusion and exclusion criteria are listed in Table 2.

**Table 2.** Inclusion and exclusion criteria.

Inclusion criteria
1. New clinical stroke diagnosis that occurred 7–60 days (inclusive) prior to randomisation
2. Age ≥18 years
3. ARAT ≤46 and FMUE hand sub-section ≤7 due to stroke
4. Capacity to consent to study participation with or without aphasia
5. Identified during stroke index admission with consent, baseline assessment and randomisation occurring as an inpatient or within 2 weeks of discharge home
6. Considered eligible to use a SaeboGlove at consent/baseline assessment:
▪ Reduced active range of digital extension with wrist held passively in full extension at consent/baseline
▪ At least 5° passive wrist extension with fingers held passively in full extension
▪ Nil to minimal digital contractures (5–10° accommodated)
▪ Some initiation of gross active digital flexion (crude estimate ≥2 cm in thumb plus ≥1 other digit, using the tips of these digits as a reference)
▪ Modified Ashworth Scale ≤2 in wrist/fingers and considered to have consistent hand opening / closing with SaeboGlove on to enable grasp / release despite tone present
7. Considered able to don/doff a SaeboGlove and engage in independent rehabilitation with or without the help of a willing carer
8. Considered able to comply with the requirements of the protocol, including questionnaires with or without help from proxy
Exclusion criteria
1. Swelling of the paretic hand considered severe enough to cause discomfort when glove is worn
2. Other significant upper limb impairment e.g. fixed contracture, fracture within last 6 months, frozen shoulder, severe arthritis, amputation
3. Diagnosis likely to interfere with rehabilitation or outcome assessments e.g. registered blind or terminal illness
4. Participant in another intervention trial

ARAT: Action Research Arm Test; FMUE: Fugl Meyer Upper Extremity scale.

### Case ascertainment, consent and screening

Potential participants will be identified by clinical care teams. Once identified, site personnel will provide a patient information leaflet and discuss study requirements with potential participants. To check eligibility, a screening assessment will then be performed (Table 1). Eligibility will be re-confirmed at the baseline assessment if it takes place >48 hours after initial screening or if the assessor has any concerns over a change in eligibility since it was initially checked (Table 1).

### Baseline assessments

The baseline assessment will be carried out by trained site personnel, and will include assessing upper limb impairment (FMUE), perceived habitual functional upper limb use (Motor Activity Log (MAL)), degree of disability or dependence (modified Rankin Scale (mRS)), activities of daily living (Barthel Index (BI), quality of life (Stroke Impact Scale (version 3.0, UK) (SIS), EuroQuol (EQ-5D-5L), upper limb pain intensity (visual analogue scale (VAS)), NHS and social services resource use pre-stroke and current upper limb treatment (Table 1). Details on each measure are given in the online Supplementary material.

### Randomisation

A central online randomisation service will be used, developed and maintained by the Robertson Centre for Biostatistics, University of Glasgow. Participants will be allocated, on a 1:1 basis, to receive 6 weeks of SaeboGlove self-directed, repetitive, functional-based practice plus usual NHS care or 6 weeks of usual NHS care. A minimisation algorithm (with a small random element) will be applied, designed to maintain balanced allocations with respect to study site, time since stroke (≤1 month, >1 month) and severity of upper limb function (ARAT 0–10, 11–28, 29–46). When randomised, only nominated unblinded site personnel will be emailed with group allocation details to progress blinded tasks.

### Interventions

#### Usual care

All participants will receive usual NHS care. Usual care involves an evidence-based approach tailored to each individual’s needs, and is based on National Clinical Guidelines that recommend a minimum of 45 minutes of physiotherapy and occupational therapy, five days per week.^9,10^ Therapy teams will be asked to record the usual care they provide during the intervention period on a study specific form (content, dose (time/movement repetitions), achievement of recommended 45-minute duration).

#### Intervention group

First use of the SaeboGlove should be within 48 hours of randomisation and must be within one week of the baseline assessment.

Participants in the intervention group will be given a SaeboGlove to use for 6 weeks. A therapist (physiotherapist/occupational) from the study team, trained in how to measure, fit and use the SaeboGlove will train participants and any assisting carers (informal or Health Care Worker) how to don/doff the glove and establish an individualised self-directed, repetitive, functional-based practice training programme involving grasping/releasing. A detailed description of the intervention used is shown using the Template for Intervention Description and Replication (TIDieR) Checklist^26^ provided in the Supplement. All therapists will have observed at least one SaeboGlove therapy session with a therapist with over 3 years’ experience in its use. Therapists will encourage participants to carry out their training programme daily and will provide a review session during 4 of the 6 weeks to assess progress and re-define their individualised upper limb treatment plan. Participants and their therapist will agree shared intensity goals for their daily practice (number of hand opening movements to aim to perform) at each review. If a participant is discharged during the intervention period, they will travel back to their local hospital to attend remaining review appointments. If this is not possible then advice will be provided by telephone.

#### Control group

Control participants will also receive a review with a therapist (physiotherapist/occupational) during 4 of the 6 weeks to assess progress and re-define their usual care individualised upper limb treatment plan and goals.

After the week 14 outcome assessment is completed, usual care participants will be offered 6 weeks of SaeboGlove self-directed, repetitive, functional-based practice. This is to minimise attrition in the control group. Data collected during this 6 week period will not be a part of the formal efficacy analysis.

#### Hand and arm rehabilitation booklets

All participants will be given a Hand and Arm rehabilitation exercise booklet to encourage self-management by recording and monitoring active upper limb therapy time during weeks 1–14 (Table 1). Site personnel will explain the booklets to them and phone them weekly to remind them to complete their rehabilitation booklet.

### Outcome assessments

Assessments will be performed at 6 and 14 weeks (range 5–9 weeks and 13–17 weeks respectively) after randomisation (Table 1) by trained site personnel. These assessments will include measures with established validity and reliability. As upper limb functional recovery is a recognised research priority for stroke survivors,^12^ the primary outcome is upper limb function measured by the ARAT at 6 weeks post randomisation. The secondary outcomes are ARAT at 14 weeks post randomisation, and FMUE, VAS, MAL, BI, mRS, SIS (full, plus hand domain only) and EQ-5D-5L at 6 and 14 weeks. Resource use will be recorded at 14 weeks also.

At 6 months a telephone follow-up will occur. This will include the EQ-5D-5L, MAL, mRS and resource utilisation. These are exploratory outcomes for economic evaluation.

All outcome assessors will receive training on the assessment before conducting any assessments. This will include face to face training, provision of an accompanying training manual and independent co-scoring of each outcome measure a minimum of 3 times with a researcher with over 5 years’ scoring experience.

### Blinding and masking of treatment allocation

Outcome assessments will be conducted by a blinded assessor. They will be asked to record if they have been unblinded at each outcome visit to enable potential bias to be reported. Given the interventions involved, it is not possible to conceal group allocation from participants or their therapist. Participants, therapists and the clinical team will be asked not to let outcome assessors know which treatment group participants are allocated to.

At the baseline assessment all participants will be given an identical small therapy box with a cardboard insert weighing the same as a SaeboGlove, and a Hand and Arm rehabilitation booklet specific for control participants (Table 1). The box will have a note in it thanking them for their participation in the SUSHI trial, asking them to bring the box with their Hand and Arm Therapy Booklets to their future study visits and to not discuss the contents of the box or the treatment group they are placed in with outcome assessors.

When participants in the intervention group are given a SaeboGlove, it will be left in their therapy box in exchange for the cardboard insert which their therapist will remove. The Hand and Arm rehabilitation booklet previously provided will also be swapped for a booklet specific for the intervention group (Table 1). All therapy booklets look identical from the outside and would need to be opened and studied to reveal group allocation. Regardless of group allocation, therapy booklets, and all case report forms used for the recording of usual care and upper limb therapy reviews will appear identical externally and will not reveal group allocation unless opened and studied. These measures will help keep outcome assessors blinded.

After the week 14 visit, SaeboGlove therapy can be offered to eligible controls, but outcome assessors should remain blinded.

### Statistical analysis

The Robertson Centre for Biostatistics within the Glasgow Clinical Trials Unit will provide statistical support to the trial. A statistical analysis plan will be agreed and made available before completion of enrolment.

Analysis will be performed on an intention to treat basis. All efficacy measures will be compared between randomised groups using linear regression (or other appropriate regression method), adjusting for the baseline value of the outcome, and minimisation variables. If statistical models fail to converge, then a reduced level of adjustment may be applied. Data may be transformed prior to analysis to satisfy statistical modelling assumptions. If no suitable regression model can be identified, then groups will be compared using a stratified Wilcoxon test (van Elteren test).^27^

### Missing data

Missing data will not be imputed for the main statistical analyses. The sensitivity of analysis results may be assessed under alternative assumptions regarding missing data, and/or through the use of multiple imputation techniques.

### Sample size calculation

A mean change in ARAT score of 10 points is assumed in the control group.^23^ A mean difference between groups of 9 points is being used for our sample size calculation based on our pilot data where participants improved by 19 points (standard deviation 13.5 points).^19^ A sample size of 49 per group will detect this difference with 90% power at a 5% significance level. Allowing for 11% attrition, we will randomise up to 110 participants to achieve this number with a 6-week outcome measure.

### Safety monitoring and analysis

Predicted adverse device effects will include discomfort in the upper limb considered to be device related. The safety of SaeboGlove self-directed, repetitive, functional-based practice and usual care will be evaluated by examining the occurrence of all adverse device events (ADEs), serious adverse events (SAEs), serious adverse device events (SADEs) and unexpected serious adverse device events (USADEs) until the 14 week visit.^28^ The occurrence of all such events will be checked at all study visits. Additionally, participants will be encouraged to report them between visits. Participants undergoing SaeboGlove therapy who have upper limb discomfort of a degree sufficient to compromise function will have study therapy stopped. Such participants will be allowed to resume therapy should they wish and if their upper limb discomfort improves, provided this is within the planned 6-week intervention period.

### Economic analysis

A within-trial cost-effectiveness analysis will be performed from the perspective of NHS and Social Services resource use. Costs will be calculated using routine data sources and data from a resource use questionnaire administered at baseline, 14 weeks and 6 months (further detail available in Supplement). Health outcomes will be expressed as quality adjusted life years using population tariffs and responses to the EQ-5D-5L at baseline, 6 and 14 weeks and 6 months (Table 1).

### Process evaluation

To aid implementation an embedded process evaluation will be performed involving the two currently approved NHS Health Boards. Semi-structured interviews will be performed with a purposive sample of at least six participants in the intervention group, four carers and four therapists (two physiotherapists and two occupational therapists) from each Board (further detail available in Supplement).

### Data management and clinical monitoring

All personal information will be collected, stored and processed in accordance with the General Data Protection Regulation (2018). Each person will be identified on case report forms only by their study number. Site personnel will record data on paper source forms and enter data into an electronic database.

A trial steering committee and a patient and carer research advisory group has been convened and will meet annually. Further details on these groups can be found in the Supplement. A Data Monitoring Committee has not been convened.

This study will be audited by designated representatives of the Sponsor, NHS GG&C, according to their audit processes.

### Dissemination

The results of this trial will be presented at research conferences and be published in peer-reviewed journals. A lay summary will be given to those participants who wish to receive it (participants will be asked at their last study visit). Anonymised data will be stored on a research repository called Enlighten for 10 years from the time of last data access and will be shared with other organisations or universities to carry out research to improve scientific understanding.

## Discussion

Impaired active digital extension is the most common motor impairment after stroke. It reduces hand opening, hindering participation in self-directed and repetitive functional-based rehabilitation recommended in national clinical guidelines, predicts poor upper limb recovery and may contribute significantly to the poor upper limb prognosis experienced after stroke. Evidence-based interventions for this group have yet to be identified. The SaeboGlove is a rehabilitation aid that assists hand opening, enabling greater access to self-directed, repetitive, functional-based practice and has been found to be a safe, feasible and acceptable intervention early after stroke. A high-quality definitive trial is now needed to assess the effectiveness and role of the SaeboGlove in the treatment of the stroke affected hand and arm in the NHS. SUSHI is a multicentre RCT to determine whether self-directed, repetitive, functional-based practice involving a SaeboGlove improves upper limb function early after stroke when compared to usual NHS care. The results from the trial will provide evidence on the clinical and cost effectiveness of SaeboGlove self-directed, repetitive, functional-based practice.

## Supplemental Material

sj-pdf-1-eso-10.1177_23969873211036586 - Supplemental material for SaeboGlove therapy for upper limb disability and severe hand impairment after stroke (SUSHI): Study protocol for a randomised controlled trial Click here for additional data file. Supplemental material, sj-pdf-1-eso-10.1177_23969873211036586 for SaeboGlove therapy for upper limb disability and severe hand impairment after stroke (SUSHI): Study protocol for a randomised controlled trial by Jen Alexander, Peter Langhorne, Lisa Kidd, Olivia Wu, Alex McConnachie, Frederike van Wijck and Jesse Dawson in European Stroke Journal

## Participating sites and principal investigators

NHS GGC

- **Queen Elizabeth University Hospital: Prof Jesse Dawson**, Institute of Cardiovascular and Medical Sciences, College of Medical, Veterinary & Life Sciences, University of Glasgow, Queen Elizabeth University Hospital, Glasgow, UK
- **Glasgow Royal Infirmary Hospital: Dr Terry Quinn**, Glasgow Royal Infirmary, 84 Castle Street, Glasgow, UK.
- **Stobhill Hospital: Dr Christine McAlpine,** Glasgow Royal Infirmary, 84 Castle Street, Glasgow, UK
- **Royal Alexandra Hospital, Dr Felicity Boyce,** Corsebar Road, Paisley, Renfrewshire

- **University Hospitals Monklands, Wishaw and Hairmyres, Dr Mark Barber**, Stroke MCN Office, Locality Offices, Hospital Street, Coatbridge, UK
