# Supplementary material for: SaeboGlove therapy for upper limb disability and severe hand impairment after stroke (SUSHI): Study protocol for a randomised controlled trial
Source: Eur Stroke J. 2021 Aug 1;6(3):302–10. doi: 10.1177/23969873211036586 (PMC8564154; doi:10.1177/23969873211036586)
Supplement: sj-pdf-1-eso-10.1177_23969873211036586 - Supplemental material for SaeboGlove therapy for upper limb disability and severe hand impairment after stroke (SUSHI): Study protocol for a randomised controlled trial [file sj-pdf-1-eso-10.1177_23969873211036586.pdf]

Supplemental materials

Template for Intervention Description and Replication (TIDieR) Checklist for the SUSHI trial intervention treatment

Item	Intervention
1. **Brief name:** Provide the name or a phrase that describes the intervention.	Help for Hands EMPOWER Programme
2. **Why:** Describe any rationale, theory, or goal of the elements essential to the intervention.	The Help for Hands EMPOWER Programme involves SaeboGlove self-directed, repetitive, functional-based practice. Rationale for this therapy: National guidelines advocate repetitive, functional-based practice and self-practice activities[1, 2]. Research shows that in order to improve brain function and regain the ability to perform every day movements after stroke, hundreds of challenging movement repetitions[3-8], specific to a given every day task[9, 10] must to be practiced. Systematic review evidence shows that after stroke 1) increased intensity of repetitive, functional-based rehabilitation is known to improve functional outcomes (dose > 20 hours)[11] and 2) self-directed interventions that involve repetitive functional-based practice can improve upper limb function and independence in activities of daily living[12]. Research on dynamic hand orthoses after stroke (including the SaeboGlove[13, 14]) consists of small feasibility studies involving between 1-20 participants with moderate and severe upper limb impairment using RCT[15-18] and non-RCT[13, 14, 19-35] designs). This preliminary evidence suggests that such devices are safe, feasible and acceptable, and enable this group to perform higher numbers of functional-based movement repetitions[13, 21, 22, 34], even when self-directed[13, 21, 22, 34]. Positive trends in upper limb recovery are observed in the acute-subacute[13, 14, 24, 32-34] and subacute-chronic[15-23, 25-31, 35] phases of recovery. Essential elements of this intervention include:<br>• SaeboGlove device<br>• Increased access to repetitive, functional-based practice<br>• Increased access to self-practice opportunities<br>• Increased intensity of training (> 100 movement repetitions daily where possible)[8]
3. **What - Materials:** Describe any physical or informational materials used in the intervention, including those provided to participants or used in intervention delivery or in training of intervention providers. Provide information on where the materials can be accessed (e.g. online appendix, URL).	The **SaeboGlove** is used[36]. It consists of a glove velcroed inside a wrist splint and tensioner bands of different size/strength which span between hooks, on the SaeboGlove, over weak joints to enable variable hand opening support, essential for self-directed, repetitive, functional-based practice (Figure 1). **Hand and Arm rehabilitation exercise booklets** are used to encourage self-management[37, 38] and record active upper limb therapy time daily. Booklets provided to the intervention group will also include a paragraph highlighting the need for intensive (> 100 movement repetitions daily[8]), repetitive functional-based practice to optimise recovery, and will

	record intensity goals agreed at each review, and encourage participants to record the number of hand opening movements they perform daily during their individualised self-directed, repetitive, functional-based training programme. A **written copy of all exercises** will be provided to participants. Additionally, therapists can **video** participants performing the exercises provided on their own / their carers mobile phone / video camera if participants feel this would be helpful.
4. **What - Procedures:** Describe each of the procedures, activities, and/or processes used in the intervention, including any enabling or support activities.	**Self-practice:** Intervention group participants will receive a SaeboGlove for 6 weeks to carry out self-directed, functional-based hand and arm exercises daily, independently with or without support of a carer. They will aim to fulfil a daily intensity goal agreed with their therapist (number of hand opening movements to aim to perform).<br>**Progress reviews:** A therapist will supervise and support participants (and carers) by providing a progress review during 4 of the 6 weeks to:<br>i) assess/reassess their ability, define/redefine their treatment plan and set shared intensity goals for their daily practice<br>ii) administer them with an individualised self-directed, repetitive, functional-based practice training programme where all exercises provided involve grasping /releasing (where possible grasping should involve an object, alternatively it should involve opposition)<br>iii) train them on the intervention, specifically on how to don/doff the SaeboGlove, and on how to perform any exercises given and log daily self-practice activity (number of hand opening repetitions and minutes of active practice) against their agreed goal in their booklet<br>iv) remind them of the ultimate goal and need to increase use of their affected hand, out with therapy exercises, in everyday tasks between each review.<br>**Rehabilitation booklet:** Site personnel will try to phone participants each week to remind them to complete their rehabilitation booklet daily.<br>**The EMPOWER principles will be followed:**<br>**E**quip with the tools needed to carry out meaningful exercise involving everyday tasks (e.g. eating, drinking, self-care) - a **SaeboGlove**, and **written or videoed exercises** involving everyday objects whenever possible.<br>**M**otivate by setting shared intensity goals and logging / monitoring daily self-practice activity. Identify and overcome barriers to doing daily exercises.<br>**P**rogress review every 1-2 weeks so exercises remain challenging and progressive.<br>**O**ptimise self-management support at reviews by coaching independence in exercises, progression

	and tailoring.<br>**W**ean from SaeboGlove bands and glove for some / all exercises as able.<br>**E**ngage carer support whenever possible.<br>**R**emind of ultimate goal and need to increase use of stroke affected hand in everyday tasks.
5. **Who provided:** For each category of intervention provider (e.g. psychologist, nursing assistant), describe their expertise, background and any specific training given.	The intervention will be carried out by participants with support from 1) a trained therapist (physiotherapist or occupational therapist) and 2) a trained carer if needed.<br>The therapist is a clinical specialist in stroke care, will receive training on the intervention, be provided with an accompanying therapy guidance document and observe at least one SaeboGlove Therapy session with a therapist with over 3 years' experience in the delivery of SaeboGlove Therapy.<br>All supporting carers will be encouraged to attend review sessions with participants where they will receive training on the intervention, specifically on how to don/doff the glove, assist with the performance of any exercises and record self-practice activity.<br>Participants and carers will be encouraged to contact their therapist for further advice if they have questions/concerns.
6. **How:** Describe the modes of delivery (e.g. face-to-face or by some other mechanism, such as internet or telephone) of the intervention and whether it was provided individually or in a group.	Typically, 1:1 face-to-face delivery, though on occasion, for participant convenience by telephone.
7. **Where:** Describe the type(s) of location(s) where the intervention occurred, including any necessary infrastructure or relevant features.	For 1:1 delivery: at bedside or clinic room in NHS hospital facilities.<br>By telephone: in NHS hospital facilities if inpatient, or at home if discharged.
8. **When and how much:** Describe the number of times the intervention was delivered and over what period of time including the number of sessions, their schedule, and their duration, intensity or dose.	Over the 6 week intervention period:<br>• Participants will be encouraged to fulfil their individualised, shared intensity goal daily.<br>• A progress review with a therapist will be provided every 1-2 weeks (4 occasions) at a mutually suitable time that also allows flexibility depending on anticipated rate of progress.<br>• SaeboGlove self-directed, repetitive, functional-based practice will be in addition to usual NHS care. It is assumed that usual care is based on National Clinical Guidelines[1, 2]. Therapy teams will be asked to ensure that participants receive 45-minutes of physiotherapy and occupational therapy daily for as long as required and will be asked to record the usual care they provide on a study specific form.
9. **Tailoring:** If the intervention was planned to be	Programs will be set at 1 of 3 levels:

personalised, titrated or adapted, then describe what, why, when, and how.	**Level 1:** 1-2 exercises involving grasping / releasing (with or without wrist movement).<br>**Level 2:** Level 1 **plus** a lower arm movement (supination/pronation or elbow flexion/extension) involving grasping / releasing.<br>**Level 3:** Level 2 **plus** an upper arm movement (any shoulder movement) involving grasping / releasing.<br>Participants will start at the highest level according to their ability. The difficulty level and number of exercises performed will be reduced / increased according to ability but all participants must be able to carry out at least one level 1 exercise with or without the help of a carer in order to participate in the trial.<br>As participants move from levels 1 to 3, exercises will become progressively more difficult because each increment will involve movement of more upper limb segments and joints, and this will require greater strength to lift the weight of an increased number of body segments up against gravity[39], the ability to coordinate movement over increasing numbers of joints, and the generation of higher torque forces (T) as the length of the moment arm (r) i.e. perpendicular distance between the joint (axis) e.g. wrist, elbow, shoulder and the hand carrying a given object or force (F)) in a task is gradually increased (T = F x r)[40].<br>Exercises selected will be functional-based movements identified as being meaningful to participants.<br><br>Participants will be encouraged to learn the best way for them to achieve their daily intensity goal e.g. doing exercises all at once, or spreading exercises out separated by breaks, doing more challenging exercises first before tiring, or last after warmed up. They will also be encouraged to increase the use of their affected hand in a varying number and type of everyday tasks between each review depending on ability and preference.<br><br>If the therapist feels that the SaeboGlove is no longer needed for some/all of the exercises, participants will continue to carry out an individualised self-directed, repetitive, functional-based practice training programme involving grasping/releasing based on level 3 with/without a SaeboGlove according to their changing ability. Similarly, the design of the SaeboGlove will enable assistance from tensioners to be increased / decreased according to individual ability.
10. **Modifications:** If the intervention was modified during the course of the study, describe the changes (what, why, when, and	To date there have been no modifications.

how).	
11. **How well - Planned:** If intervention adherence or fidelity was assessed, describe how and by whom, and if any strategies were used to maintain or improve fidelity, describe them.	Therapists will complete a Case Report Form after each review to document adherence at sessions. Participants will complete a Hand and Arm rehabilitation booklet to record the number of hand opening movements and minutes of self-practice they carryout daily. Data from booklets will be studied by therapists at reviews to monitor intervention adherence and take steps to optimise it.
12. **How well - Actual:** If intervention adherence or fidelity was assessed, describe the extent to which the intervention was delivered as planned.	Unable to describe until study completion

Outcome measures

The Fugl-Meyer Upper Extremity scale (FMUE) – The FMUE[41] is a stroke-specific assessment which measures upper limb impairment using three subscales (passive range of upper limb movement and pain during passive upper limb movement (24 items, 0-48), active movement impairment (33 items, 0-66) and sensory impairment (6 items, 0-12). Each item is rated on a 3-point ordinal scale (0=cannot perform, 1=performs partially and 2=performs fully) with a higher score representing a higher performance i.e. less impairment.

Action Research Arm Test (ARAT)—The ARAT[42] measures upper limb function and dexterity in people with hemiparesis. It scores 19 items grouped into four subscales (grasp, grip, pinch and gross movements) using a 4-point ordinal scale (0-3). Items within each subscale are arranged hierarchically, such that some items may be skipped if earlier items score 0. The final score ranges from 0-57, with 57 representing the best function i.e. normal function.

A rigorous and validated approach to ARAT assessment was followed. The original ARAT by Lyle (1981) is ambiguous with little instructions for scoring[42]. Consequently, a revised standardised[43], and validated[44] version with very high inter-rater and test-retest reliability[44] was used to increase precision of scoring.

Self-Report Measures Motor Activity Log (MAL-14)—The MAL-14 [45] is a structured interview used to assess self-perceived performance (Amount of use (AOU) and quality of use (QOU)) in 14 real-world UE activities. AOU and QOU are both rated using a 6-point scale (0-5). The mean for each scale is calculated and the final score ranges from 0-5 for each scale, with 5 indicating that the affected arm is used as much (AOU) and as well (QOU) as before the stroke. There are different versions of the MAL; MAL-14, MAL-30, MAL-28, MAL-14, MAL-45, LF-MAL, Grade 4/5 MAL and Turkish, Brazilian and German versions[46]. MAL-14 will be used as it has been studied earlier after stroke, during the subacute phase[47], and has less items and therefore a smaller burden.

Stroke Impact Scale (SIS) — The SIS[48] is a two-part stroke-specific questionnaire that measures health status after stroke. Part 1 scores 59 items grouped into eight subscales (strength, hand function, ADL/IADL, mobility, communication, emotion, memory and thinking, and participation/role function) using a 5-point Likert scale. Scores for each subscale range from 0-100, with the final score for part 1 ranging from 0 to 800. The hand function and ADL/IADL are most useful for measuring health in relation to upper limb recovery. Part 2: An overall recovery score (0- 100) is also included. A higher score reflects better health in both parts.

Barthel Index (BI) – The BI[49] measures performance in activities of daily living across 10 items of activities of daily living (feeding, bathing, grooming, dressing, bowel control, bladder control, toileting, chair transfer, ambulation and stair climbing). Items are rated in terms of whether individuals can perform activities independently, with some assistance, or are dependent. Scores for each item vary between 0-1, 0-2 or 0-3 with the overall score ranging from 0-20. A higher score represents a higher performance.

The Euroquol (EQ-5D-5L™) – The EQ-5D-5L™[50] is a self-reported questionnaire for the generic measurement of health. It scores five health dimensions (mobility, self-care, usual activities, pain/discomfort and anxiety/depression) using a five-level response (1-5, with 1 representing the best health). The responses from each dimension make a 5-digit health state profile which is combined into a single utility index value using population tariffs. An EQ-5D-5L index score of 1 represents full health.

Modified Rankin Scale (mRS) – The mRS[51] is a stroke-specific tool that measures degree of new disability/dependence after stroke using a subjective 6-point ordinal global outcome scale. Disability/dependence is rated based on an individual's independence in relation to pre-stroke activities (0=no stroke symptoms at all, 5= Severe disability due to stroke: bedridden, incontinent, and requiring constant nursing care and attention). Higher scores reflect greater disability/dependence.

Visual analogue scale for pain (VAS)[52] – A VAS is a tool often used to measure pain intensity. Individuals are asked to rate their pain intensity at a specified time (in the SUSHI trial this is the average intensity of their pain over the previous week) using an 11-point scale displayed on a 10 cm line, ranging from 0=No Pain to 10=Unbearable Pain. Higher scores reflect greater pain intensity.

All outcomes selected are widely used and accepted (FMUE[53], ARAT[53], MAL[53], SIS[54], BI[55], EQ-5D-5L[55], mRS[55], VAS[56, 57]), and have robust psychometric characteristics (FMUE[44, 58-61], ARAT[44, 60-63], MAL[45, 47], SIS[48, 64], BI[65, 66], EQ-5D-5L[67, 68], mRS[69], VAS[52, 56, 57]) in people with stroke.

However, it should be noted that while VAS is a reliable and valid measure in populations outwith stroke[70, 71], and intra-rater reliability is good in a stroke population[52], a large systematic bias has been found between raters[52]. Use of training and standardised assessment procedures have been recommended to overcome such differences[52]. Furthermore, stroke literature currently lacks a gold standard pain measure in patients with higher cortical (cognitive and visuospatial) deficits [56]. Data suggests that the validity in this group is improved using a vertical visual analogue scale [57, 72]. Thus, like all measures used, training and standardised assessment procedures will be utilised along with a training manual, a vertical scale will be used to measure upper limb pain intensity and the same assessor will assess repeated outcomes whenever possible.

Furthermore, while the MAL-14 has been found to be a reliable and valid post stroke measure[45, 47], data on the MAL is limited during the acute phase after stroke (< 1 month)[45, 47]. As SUSHI will include acute and subacute stroke patients, and acute stroke patients are likely to be inpatients with more limited opportunity to carry out everyday activities in a hospital environment, three additional upper limb activities (open a bottle or jar, tie lace, hobbies e.g. reading, cards etc) routinely accessible at a hospital beside will be scored in addition to the MAL-14 as per our pilot trial[13].

NHS and social services resource use questionnaire – The questionnaire is an adaptation of the Client Services Receipt Inventory by Beecham and Knapp (1992)[73-76]. Individuals are asked questions about the NHS and social services resources they use. This includes resources used in primary NHS care (e.g. general practice, nursing, therapy visits and prescription costs), secondary NHS care (e.g. inpatient stays and outpatient visits), social care (residential care home, nursing home and home care), and health related benefits.

Trial Management

Trial steering committee

This comprises an independent chair, two other independent members, the Chief Investigator, the co-investigator/lead physiotherapist and two other investigators. This committee shares the overall scientific responsibility of the study with the Chief Investigator and Sponsor, contributing expertise, oversight and guidance for the study duration.

Patient and carer research advisory group

This comprises two stroke survivors, two carers and the lead physiotherapist. The role of this group is to offer advice / feedback on any issues arising throughout the trial including any publications,

represent the wider views of people affected by reduced upper limb function after stroke and contribute to decision making and clinical implementation planning where relevant.

Process evaluation

The purposive sample used in the process evaluation represents more than 20% of the intervention group. Final numbers will be determined by saturation of themes (where no new themes in the data arise)[77] and resources permitting. Interviews will be designed to explore the views and experiences of participants having a stroke and receiving both usual NHS stroke rehabilitation and SaeboGlove Therapy. In order to enhance sample variation participants who provide written consent to be invited at study enrolment, will be selected to ensure representation of participants differing in age, time since stroke, severity of upper limb disability and those with and without supporting carers. Similarly, therapists will be selected to ensure representation of differing experience levels. Interviews will be conducted by a trained researcher at an agreed time and place (typically at the participants local hospital / therapists place of work), audio-recorded, and transcribed in an anonymous format. To minimise participation burden, some interviews may be carried out by telephone. Interviews will be analysed using thematic analysis[78].

Supplemental references

1. Intercollegiate Stroke Working Party. National clinical guideline for stroke, https://www.strokeaudit.org/SupportFiles/Documents/Guidelines/2016-National-Clinical-Guideline-for-Stroke-5t-(1).aspx (2016, accessed 04/02/2020).
2. National Institute for Health and Care Excellence. Stroke rehabilitation in adults https://www.nice.org.uk/guidance/cg162/resources/stroke-rehabilitation-in-adults-pdf-35109688408261 (2013, accessed 18/02/2020).
3. Kleim JA, Barbay S, Cooper NR, et al. Motor Learning-Dependent Synaptogenesis Is Localized to Functionally Reorganized Motor Cortex. *Neurobiology of Learning and Memory* 2002; 77: 63-77. DOI: https://doi.org/10.1006/nlme.2000.4004.
4. Kleim JA, Bruneau R, VandenBerg P, et al. Motor cortex stimulation enhances motor recovery and reduces peri-infarct dysfunction following ischemic insult. *Neurological Research* 2003; 25: 789-793. DOI: 10.1179/016164103771953862.
5. Luke LM, Allred RP and Jones TA. Unilateral ischemic sensorimotor cortical damage induces contralesional synaptogenesis and enhances skilled reaching with the ipsilateral forelimb in adult male rats. *Synapse* 2004; 54: 187-199. DOI: 10.1002/syn.20080.
6. Monfils MH and Teskey GC. Skilled-learning-induced potentiation in rat sensorimotor cortex: a transient form of behavioural long-term potentiation. *Neuroscience* 2004; 125: 329-336. DOI: https://doi.org/10.1016/j.neuroscience.2004.01.048.
7. Nudo RJ and Milliken GW. Reorganization of movement representations in primary motor cortex following focal ischemic infarcts in adult squirrel monkeys. *Journal of Neurophysiology* 1996; 75: 2144-2149. DOI: 10.1152/jn.1996.75.5.2144.
8. Carey JR, Kimberley TJ, Lewis SM, et al. Analysis of fMRI and finger tracking training in subjects with chronic stroke. *Brain : a journal of neurology* 2002; 125: 773-788. DOI: 10.1093/brain/awf091.
9. Boyd LA, Vidoni ED and Wessel BD. Motor learning after stroke: is skill acquisition a prerequisite for contralesional neuroplastic change? *Neuroscience letters* 2010; 482: 21-25. 2010/07/06. DOI: 10.1016/j.neulet.2010.06.082.

10. Perez MA, Lungholt BKS, Nyborg K, et al. Motor skill training induces changes in the excitability of the leg cortical area in healthy humans. *Experimental Brain Research* 2004; 159: 197-205. DOI: 10.1007/s00221-004-1947-5.
11. Pollock A, Farmer SE, Brady MC, et al. Interventions for improving upper limb function after stroke. *Cochrane Database of Systematic Reviews* 2014. DOI: 10.1002/14651858.CD010820.pub2.
12. Da-Silva RH, Moore SA and Price CI. Self-directed therapy programmes for arm rehabilitation after stroke: a systematic review. *Clinical Rehabilitation* 2018; 32: 1022-1036. DOI: 10.1177/0269215518775170.
13. Alexander J and Dawson J. The SaeboGlove Evaluation Trial. In: *European Stroke Journal* 2019/05/01 2019, pp.610-611. SAGE Publications.
14. Franck JA, Smeets RJEM and Seelen HAM. Evaluation of a functional hand orthosis combined with electrical stimulation adjunct to arm-hand rehabilitation in subacute stroke patients with a severely to moderately affected hand function. *Disability and Rehabilitation* 2019; 41: 1160-1168. DOI: 10.1080/09638288.2017.1423400.
15. Barry JG, Ross SA and Woehrle J. Therapy incorporating a dynamic wrist-hand orthosis versus manual assistance in chronic stroke: a pilot study. *J Neurol Phys Ther* 2012; 36: 17-24. 2012/02/23. DOI: 10.1097/NPT.0b013e318246203e.
16. Huang TY, Pan LH, Yang WW, et al. Biomechanical Evaluation of Three-Dimensional Printed Dynamic Hand Device for Patients With Chronic Stroke. *IEEE Trans Neural Syst Rehabil Eng* 2019; 27: 1246-1252. 2019/05/10. DOI: 10.1109/tnsre.2019.2915260.
17. Jeon H-s, Woo Y-K, Yi C-h, et al. Effect of Intensive Training With a pring-Assisted Hand Orthosis on Movement Smoothness in Upper Extremity Following Stroke: A Pilot Clinical Trial. *Topics in Stroke Rehabilitation* 2012; 19: 320-328. DOI: 10.1310/tsr1904-320.
18. Lannin NA, Cusick A, Hills C, et al. Upper limb motor training using a Saebo(™) orthosis is feasible for increasing task-specific practice in hospital after stroke. *Aust Occup Ther J* 2016; 63: 364-372. 2016/09/21. DOI: 10.1111/1440-1630.12330.
19. Barry JG, Saabye S, Baker D, et al. EFFECTS OF THE SAEBOFLEX® ORTHOSIS AND A HOME EXERCISE PROGRAM ON UPPER EXTREMITY RECOVERY IN INDIVIDUALS WITH CHRONIC. *pre* 2006; 30.
20. Butler A, Blanton S, Rowe V, et al. Attempting to improve function and quality of life using the FTM Protocol: case report. *Journal of neurologic physical therapy : JNPT* 2006; 30: 148-156.
21. Chang W-D and Lai P-T. New design of home-based dynamic hand splint for hemiplegic hands: a preliminary study. *Journal of physical therapy science* 2015; 27: 829-831. DOI: https://dx.doi.org/10.1589/jpts.27.829.
22. Chen J, Nichols D, Brokaw EB, et al. Home-Based Therapy After Stroke Using the Hand Spring Operated Movement Enhancer (HandSOME). *IEEE transactions on neural systems and rehabilitation engineering : a publication of the IEEE Engineering in Medicine and Biology Society* 2017; 25: 2305-2312. DOI: https://dx.doi.org/10.1109/TNSRE.2017.2695379.
23. Davenport S. Recovery within grasp? A report on a preliminary study investigating the short and medium term effects of the SaeboFlex (Functional Tone Management System) on chronic post stroke patients with residual upper limb deficit. *Synapse: journal & newsletter of the Association of Chartered Physiotherapists Interested in Neurology* 2005; Spring: 12-15.
24. Davenport S, Brogden S and Cairns M. The role of the saeboflex hand orthosis within therapy for patients with acute/sub-acute stroke-a proof of concept study. *Physiotherapy (United Kingdom)* 2011; 97: eS263. DOI: http://dx.doi.org/10.1016/j.physio.2011.04.002.
25. Doucet BMM, Joni A.. Pilot study combining electrical stimulation and a dynamic hand orthosis for functional recovery in chronic stroke. . *American Journal of Occupational Therapy* 2018; 72.
26. Farrell JF, Hoffman HB, Snyder JL, et al. Orthotic aided training of the paretic upper limb in chronic stroke: Results of a phase 1 trial. *NeuroRehabilitation* 2007; 22: 99-103.

27. Franck JA, Timmermans AAA and Seelen HAM. Effects of a dynamic hand orthosis for functional use of the impaired upper limb in sub-acute stroke patients: A multiple single case experimental design study. *Technology and Disability* 2013; 25: 177-187. DOI: http://dx.doi.org/10.3233/TAD-130374.
28. Gollega A, Renton S and Dezall S. SAEBOFLEX-innovative technique to improve arm function following acquired brain injury. *Stroke* 2012; 43: e155. DOI: http://dx.doi.org/10.1161/01.str.0000422054.44193.87.
29. Heise FK, Liuzzi G, Zimerman M, et al. Intensive orthosis-based home training of the upper limb leads to pronounced improvements in patients in the chronic stage after brain lesions. In: *World Congress of Neurorehabilitation (6th)* Vienna, 2010.
30. Pooyania S and Semenko B. Botulinum toxin type-A (BoNTA) and dynamic wrist-hand orthoses versus orthoses alone for the treatment of spastic-paretic upper extremity in chronic stroke patients. *Open Journal of Therapy and Rehabilitation* 2014; 2: 12-18. DOI: 10.4236/ojtr.2014.21003.
31. Prange GB, Nijenhuis SM, Sale P, et al. Preliminary Findings of Feasibility and Compliance of Technology-Supported Distal Arm Training at Home after Stroke. In: Jensen W. AO, Akay M. (ed) *Replace, Repair, Restore, Relieve – Bridging Clinical and Engineering Solutions in Neurorehabilitation Biosystems & Biorobotics*. Springer, Cham, 2014.
32. Rickards N, Sharma A and Prosser M. Feasibility Audit of Saeboflex in Stroke patients: Impact on recovery across the patient pathway http://habilis.pl/wp-content/uploads/2014/08/Saeboflex-A0-portrait2.pdf (2014, accessed 16/06/2020).
33. Semenko B. *Effectiveness of a dynamic wrist-hand orthosis in early outpatient rehabilitation of the upper extremity post stroke: a multiple single subject design evaluation* 2017.
34. Stuck RA, Marshall LM and Sivakumar R. Feasibility of SaeboFlex Upper-limb Training in Acute Stroke Rehabilitation: A Clinical Case Series. *Occupational Therapy International* 2014; 21: 108-114. DOI: 10.1002/oti.1369.
35. Woo Y, Jeon H, Hwang S, et al. Kinematics variations after spring-assisted orthosis training in persons with stroke. *Prosthetics and Orthotics International* 2013; 37: 311-316. DOI: http://dx.doi.org/10.1177/0309364612461050.
36. Saebo UK. SaeboGlove, https://uk.saebo.com/shop/saeboglove/ (2020, accessed 22/02/2020).
37. de Iongh A, Fagan P, Fenner J, et al. *A Practical Guide to Self-Management Support.* . 2015.
38. Parke HL, Epiphaniou E, Pearce G, et al. Self-Management Support Interventions for Stroke Survivors: A Systematic Meta-Review. *PLOS ONE* 2015; 10: e0131448. DOI: 10.1371/journal.pone.0131448.
39. Eng JJ. Strength Training in Individuals with Stroke. *Physiother Can* 2004; 56: 189-201. DOI: 10.2310/6640.2004.00025.
40. Swanson A. Basic Biomechanics: Moment Arm & Torque, https://www.aaronswansonpt.com/basic-biomechanics-moment-arm-torque/ (2011, accessed 17/11/2020).
41. Fugl-Meyer AR, Jääskö L, Leyman I, et al. The post-stroke hemiplegic patient. 1. a method for evaluation of physical performance. *Scand J Rehabil Med* 1975; 7: 13-31. 1975/01/01.
42. Lyle RC. A performance test for assessment of upper limb function in physical rehabilitation treatment and research. *Int J Rehabil Res* 1981; 4: 483-492. 1981/01/01. DOI: 10.1097/00004356-198112000-00001.
43. Platz T, Pinkowski C, van Wijck F, et al. *Arm. Arm Rehabilitation Measurement. Manual For Performance And Scoring: Fugl-Meyer Test (Arm). Action Research Arm Test. Box-And Block Test*

1st ed. Kappelrodeck, Germany: Deutscher Wissenschafts-Verlag, 2005.
44. Platz T, Pinkowski C, van Wijck F, et al. Reliability and validity of arm function assessment with standardized guidelines for the Fugl-Meyer Test, Action Research Arm Test and Box and Block Test: a multicentre study. *Clin Rehabil* 2005; 19: 404-411. 2005/06/03. DOI: 10.1191/0269215505cr832oa.

45. Uswatte G, Taub E, Morris D, et al. Reliability and Validity of the Upper-Extremity Motor Activity Log-14 for Measuring Real-World Arm Use. *Stroke* 2005; 36: 2493-2496. DOI: 10.1161/01.STR.0000185928.90848.2e.
46. Canadian Partnership for Stroke Recovery. Stroke Engine. Motor Activity Log, https://www.strokengine.ca/en/psycho/mal-psychometric-properties/ (2020, accessed 09/07/2020).
47. Hammer AM and Lindmark B. Responsiveness and validity of the Motor Activity Log in patients during the subacute phase after stroke. *Disability and Rehabilitation* 2010; 32: 1184-1193. DOI: 10.3109/09638280903437253.
48. Duncan PW, Bode RK, Min Lai S, et al. Rasch analysis of a new stroke-specific outcome scale: the stroke impact scale11No commercial party having a direct financial interest in the results of the research supporting this article has or will confer a benefit upon the author(s) or upon any organization with which the author(s) is/are associated. *Archives of Physical Medicine and Rehabilitation* 2003; 84: 950-963. DOI: https://doi.org/10.1016/S0003-9993(03)00035-2.
49. Collin C, Wade DT, Davies S, et al. The Barthel ADL Index: A reliability study. *International Disability Studies* 1988; 10: 61-63. DOI: 10.3109/09638288809164103.
50. EuroQol Research Foundation. EQ-5D-5L, https://euroqol.org/eq-5d-instruments/eq-5d-5l-about/ (2017, accessed 09/07/2020).
51. van Swieten JC, Koudstaal PJ, Visser MC, et al. Interobserver agreement for the assessment of handicap in stroke patients. *Stroke* 1988; 19: 604-607. 1988/05/01. DOI: 10.1161/01.str.19.5.604.
52. Pomeroy VM, Frames C, Faragher EB, et al. Reliability of a measure of post-stroke shoulder pain in patients with and without aphasia and/or unilateral spatial neglect. *Clinical Rehabilitation* 2000; 14: 584-591. DOI: 10.1191/0269215500cr365oa.
53. Santisteban L, Térémetz M, Bleton J-P, et al. Upper Limb Outcome Measures Used in Stroke Rehabilitation Studies: A Systematic Literature Review. *PLOS ONE* 2016; 11: e0154792. DOI: 10.1371/journal.pone.0154792.
54. Wu C-y, Huang Y-h and Lin K-c. Stroke Impact Scale 3.0 and the Stroke-Specific Quality of Life Scale. In: Michalos AC (ed) *Encyclopedia of Quality of Life and Well-Being Research*. Dordrecht: Springer Netherlands, 2014, pp.6368-6373.
55. Quinn TJ, Dawson J, Walters MR, et al. Functional Outcome Measures in Contemporary Stroke Trials. *International Journal of Stroke* 2009; 4: 200-205. DOI: 10.1111/j.1747-4949.2009.00271.x.
56. Price CI, Curless RH and Rodgers H. Can stroke patients use visual analogue scales? *Stroke* 1999; 30: 1357-1361. 1999/07/02. DOI: 10.1161/01.str.30.7.1357.
57. University of Glasgow and NHS Quality Improvement Scotland. *Best Practice Statement: Pain management following acute stroke*. 2011. Scotland.
58. See J, Dodakian L, Chou C, et al. A Standardized Approach to the Fugl-Meyer Assessment and Its Implications for Clinical Trials. *Neurorehabilitation and Neural Repair* 2013; 27: 732-741. DOI: 10.1177/1545968313491000.
59. Gladstone DJ, Danells CJ and Black SE. The fugl-meyer assessment of motor recovery after stroke: a critical review of its measurement properties. *Neurorehabil Neural Repair* 2002; 16: 232-240. 2002/09/18. DOI: 10.1177/154596802401105171.
60. Pandian S and Arya KN. Stroke-related motor outcome measures: Do they quantify the neurophysiological aspects of upper extremity recovery? *Journal of Bodywork and Movement Therapies* 2014; 18: 412-423. DOI: https://doi.org/10.1016/j.jbmt.2013.11.006.
61. Rabadi MH and Rabadi FM. Comparison of the action research arm test and the Fugl-Meyer assessment as measures of upper-extremity motor weakness after stroke. *Arch Phys Med Rehabil* 2006; 87: 962-966. 2006/07/04. DOI: 10.1016/j.apmr.2006.02.036.
62. Hsieh C-L, Hsueh IP, Chiang F-M, et al. Inter-rater reliability and validity of the Action Research arm test in stroke patients. *Age and Ageing* 1998; 27: 107-113. DOI: 10.1093/ageing/27.2.107.

63. Van der Lee JH, De Groot V, Beckerman H, et al. The intra- and interrater reliability of the action research arm test: a practical test of upper extremity function in patients with stroke. *Arch Phys Med Rehabil* 2001; 82: 14-19. 2001/03/10. DOI: 10.1053/apmr.2001.18668.
64. Lin K-C, Fu T, Wu C-Y, et al. Psychometric comparisons of the Stroke Impact Scale 3.0 and Stroke-Specific Quality of Life Scale. *Quality of Life Research* 2010; 19: 435-443. DOI: 10.1007/s11136-010-9597-5.
65. Duffy L, Gajree S, Langhorne P, et al. Reliability (inter-rater agreement) of the Barthel Index for assessment of stroke survivors: systematic review and meta-analysis. *Stroke* 2013; 44: 462-468. 2013/01/10. DOI: 10.1161/strokeaha.112.678615.
66. Quinn Terence J, Langhorne P and Stott David J. Barthel Index for Stroke Trials. *Stroke* 2011; 42: 1146-1151. DOI: 10.1161/STROKEAHA.110.598540.
67. Golicki D, Niewada M, Buczek J, et al. Validity of EQ-5D-5L in stroke. *Qual Life Res* 2015; 24: 845-850. 2014/10/28. DOI: 10.1007/s11136-014-0834-1.
68. Dorman P, Slattery J, Farrell B, et al. Qualitative Comparison of the Reliability of Health Status Assessments With the EuroQol and SF-36 Questionnaires After Stroke. *Stroke* 1998; 29: 63-68. DOI: 10.1161/01.STR.29.1.63.
69. Banks Jamie L and Marotta Charles A. Outcomes Validity and Reliability of the Modified Rankin Scale: Implications for Stroke Clinical Trials. *Stroke* 2007; 38: 1091-1096. DOI: 10.1161/01.STR.0000258355.23810.c6.
70. Seymour RA. The use of pain scales in assessing the efficacy of analgesics in post-operative dental pain. *Eur J Clin Pharmacol* 1982; 23: 441-444. 1982/01/01. DOI: 10.1007/bf00605995.
71. Wallenstein SL, Heidrich 3d G, Kaiko R, et al. Clinical evaluation of mild analgesics: the measurement of clinical pain. *British Journal of Clinical Pharmacology* 1980; 10: 319S-327S. DOI: 10.1111/j.1365-2125.1980.tb01816.x.
72. Benaim C, Froger J, Cazottes C, et al. Use of the Faces Pain Scale by left and right hemispheric stroke patients. *Pain* 2007; 128: 52-58. 2006/10/10. DOI: 10.1016/j.pain.2006.08.029.
73. Beecham J and Knapp M. *Costing psychiatric interventions*. London, UK: Gaskell 2001.
74. Patel A, Knapp M, Evans A, et al. Training care givers of stroke patients: economic evaluation. *BMJ (Clinical research ed)* 2004; 328: 1102-1102. DOI: 10.1136/bmj.328.7448.1102.
75. Patel A, Knapp M, Perez I, et al. Alternative Strategies for Stroke Care. *Stroke* 2004; 35: 196-203. DOI: 10.1161/01.STR.0000105390.20430.9F.
76. Rodgers H, Shaw L, Bosomworth H, et al. Robot Assisted Training for the Upper Limb after Stroke (RATULS): study protocol for a randomised controlled trial. *Trials* 2017; 18: 340. DOI: 10.1186/s13063-017-2083-4.
77. Francis JJ, Johnston M, Robertson C, et al. What is an adequate sample size? Operationalising data saturation for theory-based interview studies. *Psychol Health* 2010; 25: 1229-1245. 2010/03/06. DOI: 10.1080/08870440903194015.
78. Braun V and Clarke V. Using thematic analysis in psychology. *Qualitative Research in Psychology* 2006; 3: 77-101. DOI: 10.1191/1478088706qp063oa.
